# Clinical and Genetic Aspects of Juvenile Amyotrophic Lateral Sclerosis: A Promising Era Emerges

**DOI:** 10.3390/genes15030311

**Published:** 2024-02-28

**Authors:** Paulo Victor Sgobbi de Souza, Paulo de Lima Serrano, Igor Braga Farias, Roberta Ismael Lacerda Machado, Bruno de Mattos Lombardi Badia, Hélvia Bertoldo de Oliveira, Alana Strucker Barbosa, Camila Alves Pereira, Vanessa de Freitas Moreira, Marco Antônio Troccoli Chieia, Adriel Rêgo Barbosa, Vinícius Lopes Braga, Wladimir Bocca Vieira de Rezende Pinto, Acary Souza Bulle Oliveira

**Affiliations:** Motor Neuron Disease Unit, Division of Neuromuscular Diseases, Federal University of Sao Paulo (UNIFESP), Sao Paulo 04039-060, Brazil; paulo.lima.serrano@gmail.com (P.d.L.S.); wladimirbvrpinto@gmail.com (W.B.V.d.R.P.);

**Keywords:** Motor Neuron Disease, Amyotrophic Lateral Sclerosis, neuromuscular diseases, juvenile Amyotrophic Lateral Sclerosis, neurodegenerative diseases

## Abstract

Juvenile Amyotrophic Lateral Sclerosis is a genetically heterogeneous neurodegenerative disorder, which is frequently misdiagnosed due to low clinical suspicion and little knowledge about disease characteristics. More than 20 different genetic *loci* have been associated with both sporadic and familial juvenile Amyotrophic Lateral Sclerosis. Currently, almost 40% of cases have an identifiable monogenic basis; type 6, associated with *FUS* gene variants, is the most prevalent globally. Despite several upper motor neuron-dominant forms being generally associated with long-standing motor symptoms and slowly progressive course, certain subtypes with lower motor neuron-dominant features and early bulbar compromise lead to rapidly progressive motor handicap. For some monogenic forms, there is a well-established genotypic-phenotypic correlation. There are no specific biochemical and neuroimaging biomarkers for the diagnosis of juvenile Amyotrophic Lateral Sclerosis. There are several inherited neurodegenerative and neurometabolic disorders which can lead to the signs of motor neuron impairment. This review emphasizes the importance of high clinical suspicion, assessment, and proper diagnostic work-up for juvenile Amyotrophic Lateral Sclerosis.

## 1. Introduction

Amyotrophic Lateral Sclerosis (ALS) represents the main form of sporadic and inherited adult-onset Motor Neuron Disease (MND) [1]. Despite most cases of ALS occurring between the fifth and seventh decade of life, at least 10% of cases present as young-onset forms, with motor symptoms starting before age 45 [2,3,4]. A rare and expanding subgroup of such cases includes juvenile ALS (JALS), with onset of motor compromise before age 25. JALS is commonly misdiagnosed and underrecognized in clinical practice, mainly due to low disease awareness and several diagnostic misconceptions [5].

Global prevalence and incidence of JALS are still largely unknown [5]. A rare multicentric European study including data from 46 specialized ALS centers estimated a prevalence of 0.008 cases per 100,000 inhabitants with onset of symptoms before age 18, representing less than 0.1% of all ALS cases [6]. In a Portuguese cohort of patients with young-onset ALS, 14.3% of cases were related to JALS [7]. Since the introduction of Whole-exome sequencing (WES) and next-generation sequencing (NGS)-based tests in routine diagnostic work-up in the last two decades, there has been a significant increase in knowledge about pathophysiological mechanisms and a better understanding of the natural history and clinical complications of each monogenic form of JALS. Although only 40% of JALS cases have an identifiable genetic basis, more than 20 distinct genetic loci have been associated with JALS [5]. Most JALS cases occur as sporadic ALS (sALS) presentations but can be associated with autosomal recessive or dominant and, rarely, X-linked patterns of inheritance [5].

Given the expansion of available diagnostic methods and the development of new therapeutic modalities based on antisense oligonucleotides and viral vector platforms in gene therapy, it is essential to organize current knowledge and review the state of the art in this topic. This review article presents the main clinical, pathophysiological, genetic, and therapeutic aspects related to JALS and its main genetic subtypes.

## 2. Pathophysiology

The neuropathological involvement of JALS is not limited to upper and lower motor neuron compromise [5]. Multiple neural pathways have been associated as well as, less frequently, cognitive and affective areas and, rarely, sensory cortical regions. Several genetic subtypes related to different neuronal and glial dysfunctions have been identified [8] (Table 1). Motor neuron loss in this context results from several pathophysiological mechanisms, similarly to typical sporadic and familial ALS (fALS), including the following: (i) abnormal protein misfolding and aggregation; (ii) disturbances of autophagy; (iii) disorders of the ubiquitin-proteosome system; (iv) defects in retrograde and anterograde axonal transport; (v) mitochondrial dysfunction; and (vi) abnormal neuronal metabolic function with accumulation of neurotoxic compounds, such as reactive oxygen species, sorbitol, or other toxic intermediates [1,8,9,10]. There is important pathophysiological and genetic overlap of JALS with several other inherited neurological disorders, including Hereditary Spastic Paraplegia (HSP), axonal Charcot-Marie-Tooth disease (CMT), non-5q Spinal Muscular Atrophy (SMA), autosomal recessive cerebellar ataxia, and inherited metabolic disorders [11,12,13,14]. Glial cell dysfunction seems also to contribute in JALS pathogenesis, especially in rare cases associated with *SOD1* variants [5]. However, the scientific evidence base obtained through the observation of the monogenic basis seen in patients with JALS indicates a possible lesser importance of neuroinflammatory and neurotoxic mechanisms, and less pronounced role of microglial activation in the pathogenesis of JALS [5,8,9]. Toxic (i.e., radiation, pesticides, solvents, β-methylamino-L-alanine, methylphenyltetrahydropyridine, heavy metals, immunization), infectious (i.e., retroviruses, herpesviruses), and environmental and behavioral factors (i.e., dietary factors, low polyunsaturated fatty acid consumption, strenuous physical activity, athleticism, repeated head trauma, electromagnetic field exposure in occupation, military veterans) have been associated with ALS [1,3,9], and may potentially participate in JALS as potential trigger mechanisms when associated with genetic predisposition [1,5].

## 3. Clinical Presentation

The clinical picture in typical JALS presentation is dominated by symmetric or asymmetric upper and lower motor neuron signs with variable rates of motor progression and bulbar compromise. Almost pure upper motor neuron compromise may also be observed, presenting with marked spasticity, brisk tendon reflexes, clonus, and extensor plantar responses [9], mimicking features of HSP and Primary Lateral Sclerosis [11,12,15] (Table 2). Pure lower motor neuron involvement may also be identified, presenting with amyotrophy, weakness, fasciculation, hypotonia, and reduced or absent tendon reflexes [9], similarly to 5q and non-5q SMA and Progressive Muscular Atrophy [12,14,15,16] (Table 2). Significant cognitive compromise and autonomic disturbances are rare, and Frontotemporal Dementia (FTD) has not been directly correlated with JALS.

Ben Hamida’s classification of the JALS phenotype (1990) includes the three main groups of patients in clinical practice: (i) Group 1: distal upper limb amyotrophy form, evolving with bilateral pyramidal signs, spastic paraparesis and bulbar involvement; (ii) Group 2: late childhood- or juvenile-onset spastic paraparesis form with peroneal amyotrophy, sparing the bulbar region; and (iii) Group 3: childhood-onset spastic pseudobulbar form with spastic paraparesis [17].

The most common genetic basis associated with JALS includes variants in *FUS*, *ALS2*, *SETX,* and *SPG11* genes [5]. However, several other genes have been linked to JALS based on single case reports or case series (Table 1) [18,19,20,21,22,23,24,25,26,27,28,29,30,31,32,33,34,35,36,37,38,39,40,41,42,43,44], particularly *SIGMAR1*, *SOD1*, *SPTLC1*, *UBQLN2*, and recently *SORD* [18,19,20,21,22,23,24,25,26,27,28,29,30,31,32]. A detailed pedigree chart is important during analysis of family history and pattern of inheritance. Founder effects of variants and parental consanguinity are phenomena observed in several genetic subtypes of JALS. An autosomal recessive pattern of inheritance is observed more frequently in consanguineous families, and has been observed in patients with variants in *ALS2* [5], *SPG11* [33], *SIGMAR1* [20,22], *ERLIN1* [27], *VRK1* [34], *GNE* [26], *DDHD1* [25], and *SYNE1* genes [31]. An autosomal dominant pattern of inheritance and apparently sporadic “de novo” cases are commonly seen with variants in *FUS* [35], *SETX* [36], *SOD1*, *SPTLC1* [29], *SPTLC2* [37,38], *TRMT2B* [39], *BICD2* [37], and *TARDBP* genes. Founder effect may play a role in specific higher frequency rates [38,40], such as with *SETX* variants in families from Maryland, United States. An X-linked pattern of inheritance is highly suggestive of rare *UBQLN2* pathogenic variants [21], despite being exceptionally seen also in *TRMT2B* variants [39]. *FUS* and *SOD1* pathogenic variants represent the most common monogenic familial JALS with global distribution, despite most JALS cases occurring as sporadic “de novo” variants [38,41,42]. There is still a lack of genetic data regarding the monogenic basis of JALS in other populations, such as Latin Americans and Africans [44,45,46].

The genetic basis associated with the identifiable monogenic forms of JALS differs from the pattern observed in sporadic and familial forms of ALS globally and regionally. Sporadic and familial forms of typical ALS are related to the greater occurrence of hexanucleotide repeat expansions in the *C9orf72*, and pathogenic variants in the *SOD1*, *TARDBP* and *FUS* genes [1,8,9,10]. There is, however, the influence of local epidemiological factors and founder effects related, respectively, to the lower and higher frequency of distinct genetic basis in specific populations. Therefore, the high frequency of the pathogenic variant p.Pro56Ser in the *VAPB* gene in the Brazilian population stands out, as well as the low frequency of expansions in *C9orf72* in populations of Asian origin [1,8,9]. The potential role of founder effects in the context of JALS is not well established [5].

**Table 1:** Summary of the main clinical, epidemiological, neuroimaging and genetic aspects associated with juvenile ALS (JALS) * [5,18,19,20,21,22,23,24,25,26,27,28,29,30,31,32,33,34,35,36,37,38,39,40,41,42,43,44].

JALS Type	Epidemiology	Clinical and Neuroimaging Features	Genetics (*Locus;* Inheritance)	Allelic Disorders
JALS type 1	Second decade of life; global distribution (rare).	LMN-dominant, asymmetric; rapidly progressive. Neuroimaging: non-specific features.	*SOD1* (21q22.11); “de novo” (most cases), AD.	STAHP (AR).
JALS type 2	First/second decade of life; global distribution (more common in familial aggregates and consanguinity).	Slowly progressive spastic paraparesis/quadriparesis, pseudobulbar affect, facial spasticity, dysarthria, scoliosis; dystonia in some cases. Neuroimaging: cortical and spinal cord atrophy.	*ALS2* (2q33.1); AR.	Juvenile PLS; IAHSP.
JALS type 4	Second/third decade; global distribution; founder effect in Maryland, USA.	Slowly progressive distal amyotrophy of the lower and upper limbs; variable pyramidal release signs; variable cerebellar ataxia. Neuroimaging: non-specific features.	*SETX* (9q34); AD.	Ataxia-oculomotor apraxia type 2; Non-5q SMA with pyramidal release signs.
JALS type 5	Second/third decade; global distribution.	Slowly progressive spastic paraparesis/quadriparesis, bulbar involvement; cognitive and psychiatric disturbances; variable autonomic compromise; progressive amyotrophy during clinical course. Neuroimaging: cortical atrophy; thin corpus callosum; leukoencephalopathy (variable).	*SPG11* (15q21.1); AR.	SPG11; CMT type 2X.
JALS type 6	First-third decade of life; global distribution.	LMN-dominant with early bulbar symptoms; rapidly progressive (most cases); variable cognitive dysfunction, tremor, and myoclonus. Neuroimaging: frontal cortical atrophy; corticospinal tract signal change.	*FUS* (16p11.2); “de novo” variant (most); AD.	Hereditary essential tremor type 4; FTD.
JALS type 10	Second/third decade of life; very rare.	Rapidly progressive brachial paraparesis with distal predominance evolving to quadriparesis. Neuroimaging: non-specific changes.	*TARDBP* (1p36.22); “de novo” variant; AD.	Familial ALS type 10; FTD.
JALS type 15	Second decade of life; very rare.	Slowly progressive spastic quadriparesis; cognitive dysfunction or FTD. Neuroimaging: non-specific changes (most cases).	*UBQLN2* (Xp11.21); XLD.	FTD.
JALS type 16	First/second decade of life; rare, global distribution.	Slowly progressive brachial paraparesis, evolving to quadriparesis. Neuroimaging: non-specific changes.	*SIGMAR1* (9p13.3); AR.	AR distal SMA type 2.
6p25 and 21q22 related JALS (Utah) **	First decade of life; single consanguineous family (Utah, USA).	Slowly progressive spastic paraparesis/quadriparesis; eyelid ptosis, late bulbar involvement; dysarthria; scoliosis; gynecomastia. Neuroimaging: non-specific changes.	Unknown monogenic basis; AR.	Unknown allelic disorders.
*ERLIN1*-related JALS	Second decade of life; very rare.	Slowly progressive spastic quadriparesis, with associated motor neuronopathy. Neuroimaging: non-specific changes.	*ERLIN1* (10q24.31); AR.	SPG62.
*DDHD1*-related JALS	Second decade of life; very rare.	Progressive spastic quadriparesis with distal amyotrophy, with associated sensory neuronopathy. Neuroimaging: non-specific changes.	*DDHD1* (14q22.1); AR.	SPG28.
JALS with dementia **	First/second decade of life; single Dutch family (consanguineous).	Slowly progressive spastic paraparesis; distal amyotrophy of the hands; late bulbar involvement; severe intellectual disability, cognitive decline. Neuroimaging: unavailable (description in 1968).	Unknown monogenic basis; AR.	Unknown allelic disorders.
*SORD*-related JALS	Third decade of life; very rare.	Progressive distal amyotrophy, postural tremor, sensorineural hearing loss; scoliosis. Neuroimaging: non-specific changes.	*SORD* (15q21.1); AR.	Distal SMA, axonal CMT.
*SPTLC1*-related JALS	First/second decade of life; global distribution (rare).	Slowly progressive spastic paraparesis/quadriparesis with bulbar involvement; failure to thrive; variable sensory neuronopathy. Neuroimaging: non-specific changes.	*SPTLC1* (9q22.31); AD, “de novo” variant.	HSAN type IA.
*SPTLC2*-related JALS	Early-childhood-onset (<4 years); global distribution (rare).	LMN and UMN disease, tongue fasciculation; most cases with significant bulbar involvement (mainly dysphagia) and non-invasive ventilation support. Prior history of speech delay. Neuroimaging: normal brain and spine studies.	*SPTLC2* (14q24.3); AD; “de novo” variant.	HSAN type IC.
*SYNE1*-related JALS	Second decade of life; rare.	Progressive distal amyotrophy, dysarthria, dysphagia, cognitive decline, mild cerebellar ataxia (variable), sensory neuronopathy. Neuroimaging: mild cerebellar atrophy.	*SYNE1* (6q25.2); AR.	AR cerebellar ataxia with retained reflexes; Emery-Dreifuss muscular dystrophy type 4; Arthrogryposis multiplex congenita type 3.
*GNE*-related JALS	Second/third decade of life; very rare.	Progressive distal amyotrophy of the lower limbs; bulbar and axial involvement. Neuroimaging: non-specific changes.	*GNE* (9p13.3); AR.	Nonaka distal myopathy; Sialuria.
*C19orf12*-related JALS	First decade of life; rare.	Progressive spastic paraparesis, global amyotrophy, anarthria, pseudobulbar affect; some cases with onset as floppy baby. Neuroimaging: non-specific changes (early stages); late iron deposition.	*C19orf12* (19q12); AR.	SPG43; NBIA type 4.
*CLEC4C*-related JALS	Second decade of life; very rare.	Progressive asymmetric quadriparesis with brisk reflexes, amyotrophy and other pyramidal release signs. Neuroimaging: unknown (not reported).	*CLEC4C* (12p13.31); “de novo” variant, AD.	Increased general risk for Psoriasis and Systemic Erythematosus Lupus.
*VRK1*-related JALS	First decade of life; very rare.	Slowly progressive spastic paraparesis; mild cognitive decline, sensory neuronopathy; late amyotrophy of the hands. Neuroimaging: non-specific changes.	*VRK1* (14q32.2); AR.	Early-onset non-5q SMA; pontocerebellar hypoplasia type 1A.
*BICD2*-related JALS	Second decade of life; very rare.	UMN-dominant-ALS with bulbar involvement, tongue fasciculation, dysarthria, and pseudobulbar affect. Neuroimaging: non-specific changes.	*BICD2* (9q22.31); “de novo” variant, AD.	SMALED syndrome types 2A and 2B.
*ATP13A2*-related JALS	Third decade of life; very rare.	UMN-dominant, late bulbar involvement. Neuroimaging: cortical atrophy; cerebellar atrophy.	*ATP13A2* (1p36.13); AR.	Kufor-Rakeb syndrome, SPG78, Neuronal ceroid lipofuscinosis type 12.
*TRMT2B*-related JALS	Second decade of life; very rare.	UMN and LMN compromise, deformities of hands and feet; unclosed eyes, eyelid opening difficulty. Neuroimaging: normal brain and spine studies.	*TRMT2B* (Xq22.1)*;* XLD	Unknown allelic disorders.

* The designation of the forms of JALS follows the numbering established in the classification updated annually by the Gene Muscle Table of the World Muscle Society (WMS; available at: URL: https://www.musclegenetable.fr) (accessed on 20 January 2024) and the nomenclature of the Online Mendelian Inheritance in Man (OMIM; available at: https://www.omim.org) (accessed on 20 January 2024). Forms that have not yet been assigned specific numbers were classified based on the official name of the related gene. ** Subtypes with unidentified monogenic basis. Legend: AD: autosomal dominant; ALS: Amyotrophic Lateral Sclerosis; AR: autosomal recessive; CMT: Charcot-Marie-Tooth disease; FTD: Frontotemporal dementia; HSAN: Hereditary Sensory and Autonomic Neuropathy; IAHSP: Infantile-onset Ascending Hereditary Spastic Paralysis; LMN: Lower Motor Neuron; PLS: Primary Lateral Sclerosis; SMA: Spinal Muscular Atrophy; SMALED: Spinal Muscular Atrophy with lower extremity dominance; SPG: Spastic Paraplegia; STAHP: Progressive Spastic Tetraplegia and Axial Hypotonia; UMN: Upper Motor Neuron; XLD: X-linked inheritance (dominant).

**Table 2:** Main differential diagnosis for juvenile ALS regarding acquired and inherited etiologies with motor neuron involvement [9,11,12,14,15,16].

Differential Diagnosis	Red Flags, Clues, and General Aspects per Group	Diagnostic Work-Up
Hereditary Spastic Paraplegia (complicated forms)	Neurological signs related to multiple neuroanatomical pathways (including dystonia, parkinsonism, optic atrophy, cerebellar ataxia); Neuroimaging studies showing thin corpus callosum; Upper motor neuron-dominant phenotype or pure involvement of the corticospinal tract (low evidence of clinically symptomatic motor neuronopathy).	Targeted NGS-based gene panel or whole exome sequencing (WES).
dHMN/distal SMA	Generally symmetric with marked involvement of the lower motor neuron, mild bulbar compromise (if present, generally occurs later in disease course); Absence of well-recognizable upper motor neuron signs at clinical examination; Common presentations with pure motor involvement involving the upper limbs.	Targeted NGS-based gene panel or WES.
Non-5q proximal SMA	Complex neurological and/or systemic phenotypes associated with motor neuronopathy (i.e., sensorineural deafness, optic atrophy, cardiomyopathy); Most cases with symmetric, slowly progressive, and proximal-dominant presentation.	Targeted NGS-based gene panel or WES.
SCA	Cerebellar ataxia as dominant phenotype; Positive family history with genetic anticipation; Olivopontocerebellar atrophy; Hearing loss in SCA36; complex neurological presentation in SCA2 (choreoathetosis, neuropathy, parkinsonism, slow saccadic pursuit) and in SCA3 (focal or generalized dystonia, parkinsonism, ophthalmoparesis, “bulging-eyes” sign).	Gene panel for SCA (highlighting genetic analysis for *ATXN2*, *ATXN3*, and *NOP56* genes). Single gene testing also possible (CAG trinucleotide repeat expansion detection)—PCR/Southern blot.
Inherited neurometabolic disorders	Several juvenile-onset presentations as non-5q SMA (i.e., riboflavin transporter defects, ceramidase deficiency, GM2 gangliosidosis) and Hereditary Spastic Paraplegia (i.e., SPG5A, SPG9A, SPG35, SPG46, arginase deficiency, adrenomyeloneuropathy, adult polyglucosan body disease); Progressive clinical course with possible intermittent acute decompensation episodes; Association with other neurologic and systemic compromise signs, such as seizures, cerebellar ataxia, axonal or demyelinating polyneuropathy, sensorineural hearing loss, myoclonus, dystonia, xanthomata, cataracts, diarrhea; Neuroimaging studies showing leukoencephalopathy, cerebellar atrophy, and cervical and thoracic spinal cord atrophy.	Targeted NGS-based gene panel for potentially treatable inherited metabolic diseases or WES. Specific enzymatic assays or measurement of specific biochemical biomarkers: Plasma cholestanol; acid ceramidase or hexosaminidase A activity levels in blood leukocytes; GBE activity in leukocytes or other tissues; Plasma very-long chain fatty acids. Screening for multisystemic involvement signs.

dHMN: distal hereditary motor neuronopathy; GBE: glycogen branching enzyme; MND: Motor Neuron Disease; NGS: next-generation sequencing; PCR: Polymerase Chain Reaction; SCA: spinocerebellar ataxia; SMA: Spinal Muscular Atrophy; SPG: Spastic Paraplegia.

**Figure 1:**
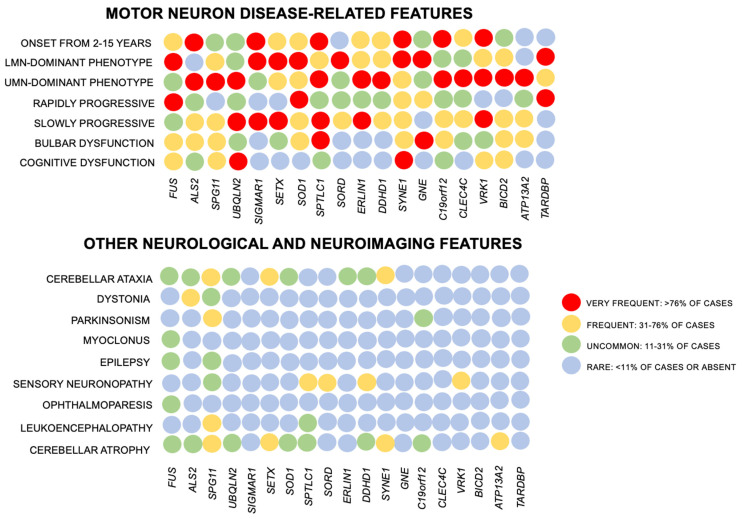
Heat map of neurological and neuroimaging features associated with JALS. Frequencies of features reported in the literature are represented by different colors, according to the specific analyzed feature and each genetic subtype.

Clinical course is generally associated with the monogenic basis involved with each JALS subtype [5,8,13]. Rapidly progressive clinical course is more commonly observed in patients with specific pathogenic variants in *FUS* and *SOD1* genes [47,48], in patients with lower motor neuron-dominant phenotypes [49,50], and in cases with prominent early bulbar compromise or in bulbar-onset JALS [35]. Slowly progressive presentation is commonly identified in cases with upper motor neuron-dominant phenotypes, mimicking HSP features, and in specific variants in *SOD1*, *ERLIN1,* and *TARDBP* genes [27,28,51,52]. A heat map with the main neurological features related to each genetic subtype of JALS is presented in Figure 1. Cognitive compromise is not a hallmark of JALS, despite the occurrence of intellectual disability and autism in *FUS* variants [53,54]. Pseudobulbar affect is most observed in *ALS2* and *SPG11* variants [33]. Cerebellar ataxia may be observed in *SETX-* and *SYNE1*-related JALS [31,41,43], while myoclonus and tremor have been described in *FUS* variants [54,55]. Diplopia and ophthalmoparesis may also be observed with *FUS* variants [56]. Furthermore, homozygous pathogenic variants in *SOD1* have recently been associated with SOD1 deficiency and a very early-onset upper motor neuron-dominant phenotype, called Progressive Spastic Tetraplegia and Axial Hypotonia (STAHP) [57]. Autonomic disturbances and variable sensory neuropathy may be identified in *VRK1*-related JALS and *SPG11* variants [5,33,34,58].

## 4. Diagnostic Work-Up and Differential Diagnosis

Clinical examination disclosing features suggestive of lower and motor neuron dysfunction is a key step during the initial assessment. Several clinical conditions share similar clinical signs with JALS and frequently represent diagnostic challenges for a definite diagnosis [9,11,12,14,15,16] (Table 2). There are no fully specific diagnostic biomarkers for the diagnostic definition of ALS and JALS. Neuroimaging studies are generally unremarkable and do not disclose specific findings. The classic “wine glass sign” associated with abnormal bilateral signal changes (hyperintensity in FLAIR and T2-weighted imaging) involving the corticospinal tracts (especially identified in coronal views) is commonly observed in JALS, especially in upper motor neuron-dominant phenotypes. Cortical and cerebellar atrophy, thin corpus callosum, and leukoencephalopathy are also detected in specific genetic subtypes (Figure 1). Needle electromyography discloses chronic denervation and reinnervation involving different spinal segments, commonly with asymmetric patterns and variable signs of abnormal spontaneous activity and acute axonal denervation (fasciculation, fibrillation potentials, positive sharp waves). Split-hand index, ALS diagnostic index, Motor Unit Number Index (MUNIX), and Motor Unit Number Estimation (MUNE) measures represent additional neurophysiological biomarkers which raise clinical suspicion of MND [59,60,61,62]. There were no previous studies which specifically evaluated the role of these neurophysiological biomarkers and indices in the diagnosis and follow-up of patients with JALS. The El Escorial criteria, the revised Airlie House criteria, the Awaji-shima criteria, and more recently the Gold Coast criteria, have been used in sporadic and familial ALS to establish levels of diagnostic certainty, following specific criteria [63,64,65,66]. In summary, the presence of signs of chronic multisegmental denervation associated with elements of acute denervation (fibrillation, positive sharp waves, fasciculations) in patients with clinical suspicion of MND allows the strengthening of diagnostic suspicion. Technical aspects relating to the application of diagnostic criteria from a laboratory and neurophysiological point of view are detailed in the original works in the literature related to the propositions of these criteria [63,64,65,66]. These criteria are currently applied also in JALS to define the presence of MND, being complemented by the onset of motor symptoms and signs before the age of 25 years, regardless of clinical severity. Nerve conduction studies may also demonstrate chronic sensory axonal polyneuropathy in cases of JALS associated with *SPG11*, *VRK1*, and *SPTLC1* genes [29,33,34,58].

Genetic testing is routinely recommended in JALS, both in sporadic and familial cases. Whole-exome sequencing or large next-generation sequencing-based multigene panels may be used in diagnostic work-up, looking for a specific monogenic basis [67,68]. Negative genetic testing results do not rule out the diagnosis of JALS. A broad genetic evaluation is essential for the following reasons: (i) providing individual and familial genetic counseling; (ii) stopping and shortening the diagnostic odyssey of patients with JALS; (iii) indicating the potential need to look for alternative differential diagnosis; (iv) providing patients and family with more reliable data about prognostic factors based on genetic aspects; and (v) including patients with specific genetic subtypes in current and future clinical trials related to viral vector-based gene therapies and antisense oligonucleotide-based therapies [6,69,70].

## 5. Treatment

Clinical trials in rare diseases during childhood and adolescence represent a great challenge due to difficulties regarding patient recruitment periods, control and placebo groups, ethical and legal aspects, outcomes, and trial designs. These are exactly the most difficult steps involved in JALS treatment [6,71]. Non-pharmacological treatment based on a specialized multidisciplinary team approach, involving physical, speech, and occupational therapists, nutritionists, psychologists, and nurses, represents the core therapy of patients with JALS. Proper management of symptomatic therapies improves the quality of life and well-being of patients in more advanced stages of the disease [1,9,72].

There are still very limited specific data regarding disease-modifying therapies for JALS. Most drugs used to treat ALS were evaluated previously in Phase 2 and 3 studies mainly for typical and sporadic forms of the disease, with a different clinical course from that observed in most cases of JALS. Thus, data on efficacy, safety, and dosage aspects of drugs potentially used in the treatment of JALS are essentially derived from clinical studies related to typical ALS, such as in the cases of Riluzole, Edaravone, and sodium phenylbutyrate with taurursodiol (tauro-ursodeoxycholic acid, TUDCA) [72] (Table 3). In such cases, for most MND-specialized centers the therapeutic indication is based on a case-by-case expert decision. In cases related to the defined single-gene base of JALS, new genetic target therapies represent great hope in the current stage of development, especially for antisense oligonucleotide therapies related to *SOD1*, *FUS,* and *ATXN2* genes [69,70,71,72,73,74,75]. Other monogenic forms of therapeutic interest include *SPTLC1*-related JALS which is amenable to oral L-serine supplementation therapy [29,76], and the rare *SORD*-related JALS which is potentially treatable with use of selective aldose reductase inhibitors [32] (Table 3).

## 6. Prognosis

Prognosis represents a complex subject during ALS management. Despite many individuals presenting with typical ALS with survival time from symptom-onset to death ranging from 20 to 48 months [9,78], more than 10% of individuals present with long-standing ALS course with more than 10 years of survival [78]. Few studies regarding the natural history of each genetic subtype of JALS are available, and most reliable data derive from several case series studies. Most JALS cases present as long-standing ALS, despite being associated with severe compromise of quality of life, marked functional capacity decline, and frequently with the need for nutritional enteral support, percutaneous gastrostomy, and permanent dependence on mechanical ventilation [52,79]. Childhood-onset, bulbar-onset, and JALS associated with complex neurological pictures are commonly more severe and have generally poor prognosis [5]. Rapidly progressive clinical course has been typically observed both in JALS types 6 (*FUS*) and 1 (*SOD1*) [5,47,48].

## 7. Conclusions

JALS represents a rare neurodegenerative disorder with several needs still unmet in clinical practice. Diagnosis is based mainly on clinical and neurophysiological aspects and supported by genetic testing, although a definite monogenic basis is not necessary for definite diagnosis. Identification of both sporadic and familial JALS cases and their involved genetic basis is essential so that genetic counseling can be provided in a timely fashion and potentially treatable etiologies can be identified earlier. Several presentations have gene therapies under investigation in current clinical trials.

## Figures and Tables

**Table 3:** Current treatment approaches and therapies under development for sALS, fALS and JALS [1,9,29,32,69,70,71,72,73,74,75,76,77].

Type of Therapy	Mechanism of Action and Characteristics of Targeted Therapies for ALS (sALS, fALS, JALS)	Phase	Level of Evidence	Reference
Non-pharmacological approaches				
- Hypercaloric enteral nutrition	Specific studies were not performed in JALS.	II	IV, RWE	[1,9]
- Early noninvasive ventilation	Early indication; dysphagia, decrease in at least 10% of body weight, or forced vital capacity < 50%.	N/A	II, RWE	[1,9]
- Physical therapy	Improvement in fatigue, quality of life and sleep.	N/A	IV, RWE	[1,9]
Riluzole	Inhibition of NMDA and kainate glutamate receptors, sodium channel block. Specific studies were not performed in JALS.	III/IV	I, RWE	[1,9]
Edaravone	Antioxidative agent, block of hydroxyl radicals. Specific studies were not performed in JALS.	III	II, RWE	[1,9]
Sodium phenylbutyrate associated with TUDCA (taurursodiol)	Phenylbutyrate: histone deacetylase inhibitor. TUDCA: neuroprotective action, anti-neuronal apoptosis. Specific studies were not performed in JALS.	III	II	[77]
AT-007 (Applied Therapeutics)	Aldose reductase-targeted inhibition: reduced conversion of glucose to sorbitol. Analysis in trials only for sensorimotor neuropathy and galactosemia. Studies were not performed in JALS.	I/II	III	[32]
L-Serine supplementation	Inhibition of β-N-methylamino-L-alanine (L-BMAA) neurotoxicity; activation of autophagic and lysosomal-endosomal pathways. Oral L-serine supplementation for JALS associated with *SPTLC1* variants.	I	IV, RWE	[29,76]
Gene-based therapies				
- *SOD1* (Tofersen)	Specific studies were not performed in JALS.	III	II	[70,75]
- *FUS* (Jacifusen; ION363)	Intrathecal antisense oligonucleotides developed to perform transcript knockdown by ribonuclease.	III *	II	[73,74]
- *ATXN2*	H-dependent degradation of mRNA.	I	IV	[69]

ALS: familial Amyotrophic Lateral Sclerosis; JALS: Juvenile Amyotrophic Lateral Sclerosis; L-BMAA: L-β-N-methylamino-L-alanine; MND: Motor Neuron Disease; N/A: not applicable; NMDA: N-methyl-D-aspartate; RWE: Real-world Evidence; sALS: sporadic Amyotrophic Lateral Sclerosis; TUDCA: Tauroursodeoxycholic acid. * Currently underway.

## Data Availability

Not applicable.

## Abbreviations

AD	autosomal dominant
ALS	Amyotrophic Lateral Sclerosis
AR	autosomal recessive
CMT	Charcot-Marie-Tooth disease
dHMN	distal hereditary motor neuronopathy
fALS	familial Amyotrophic Lateral Sclerosis
FTD	Frontotemporal Dementia
GBE	glycogen branching enzyme
HSAN	Hereditary Sensory and Autonomic Neuropathy
HSP	Hereditary Spastic Paraplegia
IAHSP	Infantile-onset Ascending Hereditary Spastic Paralysis
JALS	Juvenile Amyotrophic Lateral Sclerosis
L-BMAA	L-β-N-methylamino-L-alanine
LMN	Lower Motor Neuron
MND	Motor Neuron Disease
MUNE	Motor Unit Number Estimation
MUNIX	Motor Unit Number Index
NGS	Next-generation sequencing
NMDA	N-methyl-D-aspartate
PCR	Polymerase Chain Reaction
PLS	Primary Lateral Sclerosis
RWE	Real-world Evidence
sALS	sporadic Amyotrophic Lateral Sclerosis
SCA	spinocerebellar ataxia
SMA	Spinal Muscular Atrophy
SMALED	Spinal Muscular Atrophy with lower extremity dominance
SPG	Spastic Paraplegia
STAHP	Progressive Spastic Tetraplegia and Axial Hypotonia
TUDCA	Tauro-ursodeoxycholic acid
UMN	Upper Motor Neuron
WES	Whole-exome sequencing
XLD	X-linked inheritance (dominant)
